# Cell-free fetal DNA and spontaneous preterm birth

**DOI:** 10.1530/REP-17-0619

**Published:** 2017-12-21

**Authors:** Sara R van Boeckel, Donald J Davidson, Jane E Norman, Sarah J Stock

**Affiliations:** 1 Tommy’s Centre for Maternal and Fetal Health at the MRC Centre for Reproductive Health University of Edinburgh, QMRI, Edinburgh, UK; 2 MRC Centre for Inflammation Research University of Edinburgh, QMRI, Edinburgh, UK

## Abstract

Inflammation is known to play a key role in preterm and term parturition. Cell-free fetal DNA (cff-DNA) is present in the maternal circulation and increases with gestational age and some pregnancy complications (e.g. preterm birth, preeclampsia). Microbial DNA and adult cell-free DNA can be pro-inflammatory through DNA-sensing mechanisms such as Toll-like receptor 9 and the Stimulator of Interferon Genes (STING) pathway. However, the pro-inflammatory properties of cff-DNA, and the possible effects of this on pregnancy and parturition are unknown. Clinical studies have quantified cff-DNA levels in the maternal circulation in women who deliver preterm and women who deliver at term and show an association between preterm labor and higher cff-DNA levels in the 2nd, 3rd trimester and at onset of preterm birth symptoms. Together with potential pro-inflammatory properties of cff-DNA, this rise suggests a potential mechanistic role in the pathogenesis of spontaneous preterm birth. In this review, we discuss the evidence linking cff-DNA to adverse pregnancy outcomes, including preterm birth, obtained from preclinical and clinical studies.

## Introduction

Preterm birth (PTB), defined as delivery before 37 weeks of gestation, is the leading cause of neonatal morbidity and mortality (Goldenberg *et al*. 2008, Blencowe *et al*. 2012). Spontaneous preterm birth (spPTB) contributes to 70% of preterm births (Goldenberg *et al*. 2008). The pathogenesis of spPTB is largely unknown and worryingly, the incidence of PTB is rising globally (Blencowe *et al*. 2012). There are no effective therapies or markers to predict PTB. Indeed, only three preventative treatments have proposed potential benefit, and evidence of clinical effectiveness is conflicting (Stock & Ismail 2016). A better understanding of the pathogenesis of spPTB is urgently required to develop more effective therapies.

It is recognized that parturition is an inflammatory event. Inflammatory cells and pro-inflammatory cytokines are found in maternal and fetal tissues during labor (Christiaens *et al*. 2008, Goldenberg *et al*. 2008, Bollapragada *et al*. 2009, Cappelletti *et al*. 2016). Pro-inflammatory cytokines initiate a cascade of inflammatory mediator production, including matrix metalloproteinases and prostaglandins, which in turn lead to cervical dilation, rupture of membranes and uterine contractions (Christiaens *et al*. 2008, Goldenberg *et al*. 2008, Bollapragada *et al*. 2009). Recently, interest has grown in the potential of Cell-free fetal DNA (cff-DNA) to elicit inflammation and the parturition cascade (Phillippe 2014, Nadeau-Vallée *et al*. 2016, Herrera *et al*. 2017).

In 1997, fetal DNA was found in the maternal circulation by quantifying the male SRY gene in 43 different pregnant women (Lo *et al*. 1997). This new finding led to the development of the non-invasive prenatal test (NIPT), a test used to detect chromosomal abnormalities in the fetus by sampling of maternal blood. More recently, it has been shown that cff-DNA is increased in maternal blood in association with pregnancy complications including early pregnancy loss (Lim *et al*. 2013), preeclampsia (Contro *et al*. 2016), fetal growth restriction (Hahn *et al*. 2005, Taglauer *et al*. 2014) and preterm labor (Leung *et al*. 1998, Farina *et al*. 2005, Illanes *et al*. 2011, Jakobsen *et al*. 2012, Sifakis *et al*. 2015, Quezada *et al*. 2015, Dugoff *et al*. 2016). This has led to research into the potential for cff-DNA concentrations to be used as a biomarker for pregnancy complications (Bauer *et al*. 2006, Sifakis *et al*. 2015, Contro *et al*. 2016, Dugoff *et al*. 2016, Nadeau-Vallée *et al*. 2016). The putative mechanisms linking cff-DNA to the pathogenesis of pregnancy complications are also a new area of investigation (Scharfe-Nugent *et al*. 2012, Goulopoulou *et al*. 2016, Nadeau-Vallée *et al*. 2016, Conka *et al*. 2017).

The aim of this review is to examine the role of cff-DNA in the pathogenesis of spPTB. We will present evidence relating to (a) the biological and pro-inflammatory activities of cell-free DNA; (b) cff-DNA concentrations in the circulation of women who deliver preterm and (c) preclinical studies which examine the causative link between cff-DNA and preterm birth.

## Cell-free DNA in physiology and pathology

The human body releases cell-free DNA (cfDNA) into the circulation through cell death. In healthy people cfDNA, principally of hematopoietic origin, can be found circulating in both the plasma and serum (Jung *et al*. 2010). Indeed, circulating cfDNA may have biological functions, such as messaging functions after transcription to RNA (Jung *et al*. 2010). Although cfDNA is found free in the circulation, the majority is adherent to the surface of blood cells (Jung *et al*. 2010, Hahn *et al*. 2014). cfDNA has been shown to have a short half-life in the body and can be cleared as quickly as half an hour (Lo *et al*. 1999). Detection of cfDNA in healthy individuals is therefore normal, and a cutoff to distinguish ‘pathogenic’ from ‘normal’ values has not yet been established, as values between 6 and 650 ng/mL have been measured in healthy men (Jung *et al*. 2010). This is in part due to high intra-patient variance, but also due to different methods of cfDNA quantification that are used (Fernando *et al*. 2010, Jung *et al*. 2010, Manokhina *et al*. 2014).

High levels of cfDNA have been reported in infectious, ischemic, malignant and autoimmune diseases, obesity and during pregnancy (Jung *et al*. 2010, Dwivedi *et al*. 2012, Nishimoto *et al*. 2016). This likely relates to high levels of tissue necrosis (e.g. in sepsis) or abnormal high cell turnover in tissues (e.g. from tumors, or from the placenta in pregnancy) (Enninga *et al*. 2015).

A protective host response to ‘non-self’ or pathogenic DNA (viral or bacterial DNA) is essential for an adequate host defence response and underpins the aspects of our immune systems. However, inappropriate inflammatory responses against ‘self-DNA’ can be detrimental, and break immunological tolerance, leading to autoimmune diseases (Bauer 2006, Ori *et al*. 2017). Humans therefore have critical mechanisms to discriminate between ‘self’ and ‘non-self’-DNA. Nevertheless, in certain *in vivo* models, cfDNA originating from tumor cells and from adipocytes has been shown to elicit an inflammatory response on epithelial cells and by attracting macrophages to adipocytes, respectively (Fűri *et al*. 2015, Nishimoto *et al*. 2016). These data suggest that under certain conditions cfDNA can bypass protective mechanisms and be pro-inflammatory.

## DNA-sensing mechanisms – TLR9 and STING pathways

There are two primary DNA-sensing pathways in cells that have been linked to cfDNA sensing: the Toll-like receptor (TLR) 9 pathway and the Stimulator of Inteferon Genes (STING) pathway (Fűri *et al*. 2015).

### TLR9

**Figure 1:**
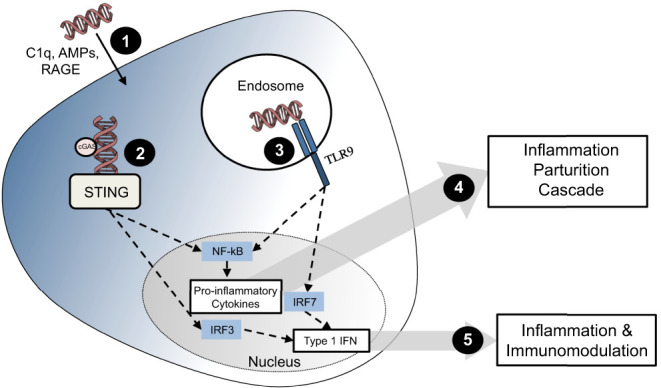
DNA sensing through STING and TLR9. (1) DNA enters the cells through a variety of mechanisms, including interactions with C1q, antimicrobial peptides (AMPs) and receptor for advanced glycation end-products (RAGE). (2) This DNA can then be sensed by binding to STING directly or by firstly biding to cyclic GMP-AMP synthase (cGAS). STING activation produces type 1 interferons through transcription factor interferon receptor factor 3 (IRF3) and, to a lesser extent, pro-inflammatory cytokines via activation of NF-κB. (3) TLR9 is found in the endosome and unmethylated DNA (CpG) or DNA with a modified back bone are typical ligands. TLR9 activation produces type 1 interferons and pro-inflammatory cytokines through IRF7 and NF-κB. (4) Pro-inflammatory cytokines are hypothesized to elicit a potent inflammatory response that can lead to the parturition cascade. (5) Type 1 interferons are known to play a role in inflammation and immunomodulation.

TLRs are pattern recognition receptors (PRRs), which activate the innate immune response when they sense damage-associated molecular patterns (DAMPs) (O’Neill *et al*. 2013). TLR9 is an intracellular receptor that senses DNA, specifically hypomethlated CpG DNA sequences found in high frequency in viral and bacterial DNA. Hypomethylated microbial DNA can be identified by membrane receptors, such as Fc Receptors, and transported to the intracellular compartment where it can be sensed by TLR9 (Bauer 2006). Under normal conditions, adult vertebrate DNA is a poor TLR9 ligand (Marsman *et al*. 2016). However, placental and fetal DNA is more hypomethylated than adult DNA and may therefore have the potential to be a TLR9 ligand (Scharfe-Nugent *et al*. 2012, Phillippe 2015). Other changes in DNA morphology can also influence TLR9 affinity, such as bending of the DNA backbone of vertebrate DNA (e.g. by nucleosomes found in cell-free DNA or binding to antibodies or antimicrobial peptides). In addition, when ‘self-DNA’ is artificially transfected into dendritic cells (DC), it can induce a TLR9 response (Yasuda *et al*. 2006). A number of mechanisms may introduce DNA into the cell cytoplasm (Fig. 1), including binding to antimicrobial peptides (human-beta defensin-3 or human cathelicidin LL-37), anti-DNA antibodies, DNA-like receptors (RAGE) or histones (Nakagawa & Gallo 2015, Marsman *et al*. 2016, McGlasson *et al*. 2017).

Successful activation of TLR9 by DNA will recruit the myeloid differentiation primary response gene 88 (MyD88). This associates with interleukin-1 receptor-associated kinase (IRAK) 4 and IRAK2 and subsequently recruits tumor-necrosis factor-α receptor factor 6 (TRAF6). This leads to the activation of pro-inflammatory signaling cascades including NF-ĸB activation, by transforming growth factor-β-associated kinase 1 (TAK1), mediated phosphorylates of IĸB (Krieg 2006, Vollmer 2006). These pathways result in changes in gene transcription and the production of pro-inflammatory cytokines (Krieg 2006, Vollmer 2006). The specificity of these responses is cell dependent, with activation of TLR9 in plasmatocytoid DC, for example, resulting primarily in interferon-α production, through recruitment of interferon regulatory factor-7 (IRF-7) (Vollmer 2006, Nakagawa & Gallo 2015).

### STING

Activation of the STING pathway has been implicated in viral, bacterial and ‘self’-DNA-mediated stimulation and is independent of TLR9 activation (Barber 2015). STING is an intracellular protein that is expressed in hematopoietic cells (including T cells, macrophages and DC) as well as endothelial and epithelial cells. DNA activates the STING pathway by binding to cyclic GMP-AMP synthase (cGAS) and the subsequent production of cyclic dinucleotides. In addition, double-stranded DNA can directly bind to STING (Fűri *et al*. 2015). Once activated, STING changes in conformation and complexes with TANK-binding kinase (TBK1). TBK1 in turn phosphorylates transcription factors IRF3 and NF-ĸB. This results in the release of IFN-β and IFN-α, and other pro-inflammatory mediators, dependent upon cell type and stimulation (Barber 2015). Similar to TLR9, DNA must be transported into the cytosol in order to activate STING and these downstream cascades (Fig. 1).

Both TLR9 and STING therefore present mechanisms by which the detection of cfDNA might be pro-inflammatory. However, there is little direct evidence that cff-DNA actually does stimulate TLR9 and whether cff-DNA can elicit inflammation through other pathways, including STING, remains unknown. As such, the effect of these pathways on spPTB remains to be determined.

## Cell-free fetal DNA

Cff-DNA originates from the placenta via cell death, likely by apoptosis in normal pregnancies (Masuzaki *et al*. 2004, Reddy *et al*. 2008, Manokhina *et al*. 2015, Nadeau-Vallée *et al*. 2016) or necrosis during infection (Hahn *et al*. 2005) in the syntiotrophoblast and cytotrophoblast layers. In addition, an association between cff-DNA and placental microparticles (such as exosomes) is clear, with various studies having found that these microparticles can release cff-DNA and demonstrating fetal DNA in, or bound to, placental microparticles (Orozco *et al*. 2008, Tong & Chamley 2015). In contrast, cfDNA (from non-fetal origin) is known to be present unbound and free in the plasma (Gold *et al*. 2015). It is however unknown how much the contribution of placental microparticles (bound or released by) contributes to the total cff-DNA found in the maternal plasma. Recently, placental extracellular vesicles have been shown to activate endothelial cells in a TLR9-dependent manner (Tong *et al*. 2017). Further clarity on the state of cff-DNA in the maternal circulation is required, as this may influence the manner in which cff-DNA can interact with immune cells and receptors (Tong & Chamley 2015).

In normal pregnancies, cff-DNA steadily increases throughout gestation (Birch *et al*. 2005) and is cleared as quickly as two hours after delivery of the placenta (Lo *et al*. 1999). An increase in non-fetal cfDNA was associated with labor, where 75.3% of the cell-free DNA is found to be maternal (quantified by whole-genome bisulfite sequencing) compared to 73.8% in term non-labor (Herrera *et al*. 2017). The percentage of cff-DNA in total cfDNA is estimated to be about 19–26.2% in normal, non-labouring pregnancies between 10 and 36 weeks (Hui & Bianchi 2011, Herrera *et al*. 2017). Pregnancies complicated with placental pathologies such as IUGR and preeclampsia have increased amounts of cff-DNA in the maternal circulation (Hahn *et al*. 2005, Reddy *et al*. 2008, Taglauer *et al*. 2014, Contro *et al*. 2016).

### Cell-free fetal DNA concentrations and preterm birth: evidence and study limitations

Within a year of the discovery of cff-DNA in the maternal circulation, high cff-DNA concentrations in maternal circulation were correlated with spPTB (Leung *et al*. 1998). In this first report, cff-DNA levels were calculated using the SRY gene quantification (whereby a quantification could be done of cff-DNA from women pregnant with a male fetus) method in 32 women with spPTB symptoms. Women who delivered preterm had significantly higher cff-DNA levels than those who did not (Leung *et al*. 1998). In a similar study, Farina and colleagues quantified cff-DNA levels of 50 women at onset of spPTB symptoms and found an association of increased cff-DNA levels and spPTB deliveries (Farina *et al*. 2005).

Subsequently, eight studies have investigated the association between cff-DNA and adverse pregnancy outcomes in asymptomatic women, with quantification from first trimester (concurrent with prenatal testing for fetal anueploides) up to 25 weeks of pregnancy (Table 1).

**Table 1:** Publications investigating the association between cell-free fetal DNA (cff-DNA) and adverse pregnancy outcomes including spontaneous preterm birth (spPTB).

Publication	Total *N* and (spPTB cases)	Study setup	Methods	Main findings
Leung *et al.* (1998)	32 (20)	Prospective cohort study to assess association with cff-DNA and spPTB	Quantification of SRY gene in maternal plasma at onset of PTB symptoms	Significantly higher *SRY* detection in women who deliver preterm (*P* = 0.042). Lower concentration of cff-DNA associated with successful tocolytic therapy (*P* = 0.017)
Farina *et al.* (2005)	71 (50)	Cross-sectional study of women at high risk for spPTB	Quantification of *DYS1* gene in maternal serum at onset of PTB symptoms	Higher *DYS1* detection in women who deliver preterm, by regression analysis of cff-DNA and gestational age at delivery (*P* = 0.003), significant maker when using cutoff of 1.82 MoM DYS1 gene
Bauer *et al.* (2006)	84 (7)	Prospective analysis for Cff-DNA as an indicator for adverse pregnancy outcomes	Quantifying the *SRY* gene and short tandem sequence from maternal plasma at amniocentesis (average 15 weeks) with blood sample	No significant increase in women who later delivered preterm (gestational age of 15.7 ± 0.5 at time of Cff-DNA quantification)
Illanes *et al.* (2011)	56 (14)	Case-control study to assess cff-DNA and risk of spPTB	*DYS* gene quantification from maternal plasma at 22–24 weeks in combination with a cervical length measurement	No correlation between cff-DNA levels and gestational age at delivery (*r* = −0.23; *P* = 0.07)
Stein *et al*. (2013)	611 (76)	Prospective cohort study to assess cff-DNA and adverse pregnancy outcome in low risk pregnancies	*RhD* gene quantification at 25 weeks (mean gestational age at quantification)	No significant increase in women who delivered preterm
Jakobsen *et al.* (2012)	876 (19)	Prospective cohort study to assess association with cff-DNA and spPTB	*RhD* gene quantification at 25 weeks of gestation	Strong association between cff-DNA levels above the 95th centile and subsequent spPTB (odds ratio of 6.3; 95% confidence interval: 1.9–20.9)
Poon *et al.* (2013)	1949 (20)	Prospective cohort study to assess cff-DNA and adverse pregnancy outcome	Chromosome selective assay at 11–13 weeks of gestation	No significant increase in regression analysis (20 deliveries <34 weeks of gestation *P* = 0.46)
Quezada *et al.* (2015)	3169 (103)	Cross-sectional study to assess cff-DNA and prediction of spPTB	Fetal Fraction quantified at 10–14 weeks with chromosome selective assay	No significant increase in women who deliver preterm
Dugoff *et al.* (2016)	1653 (119)	Retrospective cohort study at increased risk for aneuploidy	Methylation method and regional read depth counts from autosomes generated by whole-genome low coverage massively parallel single-end sequencing at 10–20 weeks	Elevated fetal fraction levels at 14.1–20 weeks were significantly associated with incidence of preterm birth (adjusted odds ratio, 4.59; 95% confidence interval, 1.39–15.2)
Thurik *et al*. (2016)	527 (49)	Nested case-control study to assess cff-DNA and adverse pregnancy outcome	Quantification of *DYS14* gene at 8–14 weeks of gestation	No association with spPTB (49, *P* = 0.19)

Four studies have quantified cff-DNA in early pregnancy (<16 weeks average gestational age at cff-DNA quantification) (Bauer *et al*. 2006, Poon *et al*. 2013, Quezada *et al*. 2015, Thurik *et al*. 2016). These included a total of 5729 women with 159 spontaneous preterm deliveries (defined as either <34 (Poon *et al*. 2013) or <37 weeks (Bauer *et al*. 2006) of gestation at delivery (Quezada *et al*. 2015, Thurik *et al*. 2016)). No association between cff-DNA levels and subsequent spPTB was seen in any of these studies; however, three of the four studies only looked for an association between cff-DNA and overall adverse pregnancy outcomes and were not sufficiently powered to specifically detect an effect of cff-DNA on spPTB. Two of these studies found associations between maternal parameters and cff-DNA. Thurik *et al.* found an inverse association between cff-DNA and maternal obesity and smoking (Thurik *et al*. 2016). Bauer *et al* found an association between increased cff-DNA and fetal abnormalities, HELLP syndrome, IUGR, gestational diabetes (Bauer *et al*. 2006, Thurik *et al*. 2016). An important potential confounder in these associations was gestational age at sampling – as neither of these studies adjusted for gestational age in their calculated associations with pregnancy outcomes, despite the well-recognized relationship between gestational age and amount of cff-DNA (Birch *et al*. 2005). In the largest study of early gestational cff-DNA, Quezada and colleagues were limited to the gestational age of 11–13 weeks and also found no association between cff-DNA and spPTB <34, 34–37 and <37 weeks, although the quantification used combined cff-DNA and cfDNA levels to quantify the fetal fraction (Quezada *et al*. 2015).

Quantification of cff-DNA levels at later points in gestation have provided conflicting evidence regarding the relationship between cff-DNA and spPTB. Illanes and colleagues investigated the predictive value of cff-DNA for spPTB in 56 women by quantifying the DYS gene (a gene found on the Y chromosome) at 22–24 weeks of gestation in combination with cervical length screening. They found no correlation between cff-DNA levels and gestational age at delivery (*P* = 0.07) and showed that this was not a predictive marker in combination with cervical length measurement (Illanes *et al*. 2011), although the numbers of spPTBs were small. Stein and colleagues reached a similar conclusion after quantifying cff-DNA using RhD fetal-specific PCR in 611 low-risk pregnancies between 19 and 32 weeks of gestation. No statistically significant difference in cff-DNA levels was seen in women who developed preeclampsia (*n* = 44), IUGR (*n* = 22) or PTB (*n* = 76) compared to those who had uncomplicated pregnancies (Stein *et al*. 2013). However, no adjustment for gestational age at sampling was made. A study by Jakobsen and colleagues investigated a larger group of women, but was limited to the gestational age of 25 weeks, found a lower prevalence of spPTB (19 cases) and observed a strong association between cff-DNA levels above the 95th centile and subsequent spPTB (*P* = 0.002). This raises the possibility that gestational age at sampling might be critical. To assess this, another study directly compared the fetal fraction levels between 11–14 weeks and 14–20 weeks. This quantification calculates the ratio of cff-DNA to total cell-free DNA. In this study, only the latter group showed a significant association between high levels of cff-DNA and the likelihood of spPTB (Dugoff *et al*. 2016).

It is difficult to conduct a formal comparative analysis and make accurate conclusions of the studies described above due to the substantial heterogeneity, not only in participants, but also in the methodologies used to extract and quantify cff-DNA. Furthermore, given that here is currently no consensus on the normal range of cff-DNA at any point in gestation, adjustments for gestational age are difficult to make.

### Participants

Studies had different inclusion and exclusion criteria. Many studies were small, and not able to adjust for variables known to influence the levels of cff-DNA, which include gestational age at sample collection, intra-uterine growth restriction, preeclampsia, smoking status and obesity (Urato *et al*. 2008, Wang *et al*. 2013, Taglauer *et al*. 2014). It will be important that future studies are sufficiently well powered to enable detailed interrogation, and ideally, allow longitudinal sample collection across multiple gestational ages.

### Extraction method

The method of cff-DNA extraction is not standardized, and different methods were used in these studies, including commercial DNA extraction kits (Qiagen), quantification directly from the maternal serum or plasma and chemagic magnetic separation. Different blood tubes were used for blood collection and storage (EDTA, sodium citrate, lithium heparin), and cff-DNA was analyzed from either fresh or stored samples, and cff-DNA was quantified from either serum or plasma. Recently, these factors have shown to have an influence on the total cff-DNA yield in different fields (Fernando *et al*. 2010, Manokhina *et al*. 2014). Direct comparisons of different methodologies on the same samples and subsequent standardization of approach will be necessary to enable more effective conclusions in the future.

### Quantification

A further complication to meta-analysis is the use of different measures of DNA quantity. For example, some studies use the fetal fraction (ratio of fetal DNA to total DNA) while others measure the total amount of cff-DNA in maternal plasma (Manokhina *et al*. 2015, Dugoff *et al*. 2016). The method of quantification also varied between studies. Initial studies limited their approach to researching only pregnancies with male fetuses in order to differentiate cff-DNA (DNA from the Y chromosome) from maternal DNA. However, more recent methodologies include using placenta-specific methylation characteristics (Manokhina *et al*. 2014, Dugoff *et al*. 2016) or cff-DNA-specific short tandem repeats (Bauer *et al*. 2006). Both these methods have capacity to quantify cff-DNA irrespective of fetal sex or blood group status. Furthermore, molecular techniques used in quantification have also varied; including PCR, massively parallel single-end sequencing and chromosome selective assays. These differences have the potential to impact the yield of cff-DNA and consequently alter findings.

In summary, cff-DNA has been measured throughout pregnancy. Four out of ten studies concluded a significant increase in cff-DNA levels in women who delivered preterm when compared to women who delivered at term, when measurements were made in the 2nd and 3rd trimester or at the onset of spPTB symptoms. However, these studies have generally looked at small populations with numbers that were insufficient to confirm the prognostic ability of cff-DNA. Nevertheless, these higher levels of circulating cff-DNA detected in later gestation and in labor (including preterm labor in four studies described above), do raise the possibility of role in the onset of parturition (Birch *et al*. 2005, Phillippe 2014, 2015, Herrera *et al*. 2017). In this context, in additional to further, more definitive studies, it is important to consider potential mechanisms of action, if indeed this is indeed a causative, not simply associated, phenomenon. It has been hypothesized that cff-DNA is pro-inflammatory and can stimulate, or contribute to the stimulation of, the inflammation-parturition cascade, following detection by intracellular DNA receptors, including TLR9 (Scharfe-Nugent *et al*. 2012). Studies aiming to evaluate this mechanistically *in vivo* in the context of pregnancy and spPTB have been conducted in animals (Thaxton *et al*. 2009, Scharfe-Nugent *et al*. 2012, Sun *et al*. 2013, Lin *et al*. 2014, Conka *et al*. 2017).

## Cell-free fetal DNA as a pro-inflammatory stimulus

We found no studies that have investigated the role of the STING pathway in adverse pregnancy outcomes *in vitro* or *in vivo*. However, six studies have investigated the effects of TLR9 activation in pregnancy and parturition in mice, with varied results.

Our group has investigated the effect of time to delivery by intra-uterine injected mouse placental DNA (van Boeckel *et al*. 2017). Ultrasound-guided intra-uterine injections of 3–300 μg/dam of mouse placental DNA was administered on day 17 to wild-type C57Bl/6 mice. We found no significant decrease in time to delivery in the mouse placental DNA administered groups, while mice treated with 1 μg of LPS all delivered preterm (*P* < 0.0001), similar to previously published findings (Rinaldi *et al*. 2015). We examined the effects of cell-free placental DNA *in-vitro* using DNA extracted from a placental explant method (van Boeckel *et al*. 2017) and found that up to 500 ng/mL elicited no inflammatory response in peripheral blood mononuclear cells (PBMCs) from pregnant women. Furthermore, we compared the total amount of unmethylated CpG motifs in cff-DNA extracted from the supernatant of human placental explant culture, E-coli DNA and adult human DNA extracted from human blood. We demonstrated the E-coli had 9.1% of unmethylated CpGs compared to 0.06% unmethylated CpGs in cff-DNA and 0% of adult DNA (van Boeckel *et al*. 2017). This demonstrates that *E coli* DNA is a better TLR9 ligand compared to cff-DNA and may be a reason for the lack of pro-inflammatory properties seen in our *in vitro* and *in vivo* experiments (van Boeckel *et al*. 2017).

These findings differ from the findings in a 2012 study by Scharfe and colleagues. Scharfe and coworkers used human fetal genomic DNA, which is larger in size than cff-DNA, but their findings suggest fetal DNA is less methylated than human adult DNA (however, no comparison with *E coli* DNA was made). Using PBMCs from non-pregnant women, they observed that fetal DNA stimulation resulted in the production of the inflammatory cytokine interleukin (IL) 6 with a similar magnitude to that induced by synthetic unmethylated CpG oligonucleotides (CpG). Further activation of TLR9 was demonstrated by IΚBα degradation in both CpG and fetal DNA-stimulated PBMCs. PBMCs from pregnant women showed a significant increase in IL-6 after fetal DNA stimulation, but no comparison to CpG was made. Subsequently, pregnant C57Bl/6 mice were given a single intra-peritoneal injection of 300 μg/dam human fetal DNA between gestational days 10 and 14. There were higher rates of fetal resorption when compared to control groups or mice injected with human adult DNA. This effect was significantly reduced in TLR9-knockout mice. However, despite these findings, this study found no systemic inflammatory response, no true PTB and no *in vitro* inflammatory response of murine macrophages to fetal DNA (Scharfe-Nugent *et al*. 2012). Nevertheless, these data implicated a TLR9-mediated effect on fetal viability specific to stimulation with fetal DNA *in vivo*. TLR9 is strongly expressed in the decidua of spontaneous abortion compared to normal human pregnancies (Kang *et al*. 2015), making it possible that targeted inflammatory stimuli to the reproductive tract might elicit an inflammatory response leading to PTB. However, the finding that CpG/TLR9 simulation can cause fetal resorption and/or PTB in healthy wild-type mice has not been replicated in subsequent studies in specific genetically modified mouse models.

Thaxton and coworkers found that hypomethylated CpG DNA (CpG) caused adverse pregnancy outcomes in IL-10-deficient mice, in the context of a study investigating the safety profile of CpG as a vaccine adjuvant in pregnancy. Intraperitoneal injections of dams with 25 μg CpG on days 6 or 14 of gestation, induced significant levels of fetal resorption and/or spPTB compared to controls. However, these adverse responses were not seen in wild-type C57Bl/6 mice, indicating that IL-10 had a protective function against harmful responses to, or recognition of this hypomethylated CpG DNA. The CpG-treated IL-10-deficient mice had higher numbers of uterine neutrophils and macrophages than the control IL-10-deficient mice and higher levels of tumor necrosis factor (TNF) in the maternal circulation. Interestingly, the adverse pregnancy outcomes in the CpG-treated IL-10-deficient mice could be reversed by depleting macrophages or by neutralizing maternal TNF, suggesting a macrophage-driven mechanism, occurring in the absence of IL-10-mediated inhibition (Thaxton *et al*. 2009).

Sun and colleagues used CpG to mimic bacterial DNA and the effects this might have on spontaneous abortion and PTB, in the context of infection. They administered intra-peritoneal injections of CpG (25–400 µg/dam) on gestational days 6 or 14 to wild-type BALB/c and natural killer (NK) cell-deficient non-obese diabetic (NOD) BALB/c mice. NOD mice showed 80–90% rates to CpG-mediated PTB and fetal resorption, while the CpG-treated wild-type mice showed no adverse effects. The adverse effects could be significantly diminished using TLR9 competitive antagonist ODN 2088 or by repletion of NK cells. These data highlight the importance of NK cells and the essential role of TLR9 in the CpG-abortion and PTB pathway (Sun *et al*. 2013).

In 2014, Lin and colleagues further investigated the ‘CpG-mediated pregnancy failure’ pathway in NOD mice. They use the same model as the Sun and colleagues, and showed that administration of interleukin 10 (IL-10) or T-regulatory cells could prevent the CpG-induced PTB/fetal resorption. These data reinforce the protective importance of IL-10 in these models. In line with the previous findings, wild-type mice did not have any adverse response to the injected CpG (Lin *et al*. 2014).

The studies described above do suggest that in the context of specific impairments to the normal immune system, CpG can induce adverse pregnancy outcomes. However, the studies were not using fetal DNA (as used by Scharfe and colleagues), nor placental DNA, which is the origin of cff-DNA (Bianchi 2004). In contrast, Čonka and colleagues did include use of DNA from placental origin in a preeclampsia mouse model. They administered daily intra-peritoneal injections of different types of DNA (human fetal, mouse placental and mouse adult DNA) or lipopolysaccharide (LPS) as a positive control once a day during gestation days 14–18 in wild-type C57Bl/6 mice. Whereas LPS induced fetal resorption in all cases (*P* < 0.001), none of the DNA-injected mice showed significant decreases in litter size. In this model, they hypothesized that TLR9 activation could cause preeclampsia; however, the different types of DNA were not able to induce preeclampsia-like symptoms (Conka *et al*. 2017). This study therefore contributes to the weight of evidence that systemic exposure of a healthy mouse to mammalian DNA (irrespective of methylation status) cannot induce adverse pregnancy outcomes without additional impairments of the immune system.

In conclusion, DNA has the potential to be pro-inflammatory, through activation of TLR9 and STING pathways. However, whether it can act as a stimulus that directly induces the parturition cascade is unknown, with conflicting *in vitro* and *in vivo* data. However, given that cff-DNA is found in the maternal circulation, DNA-sensing mechanisms exist and that these may function incorrectly in pregnancy in the context of additional immune system compromise, further investigation into the pro-inflammatory properties of cff-DNA and the consequences of cff-DNA-induced inflammation in pregnancy is merited.

## Summary

In this review, we have summarized the studies that have quantified cff-DNA in the maternal blood and examined those associated with PTB. There is evidence that PTB is associated with increased cff-DNA levels after 20 weeks or with the onset of spPTB symptoms. Further investigation is needed to standardize cff-DNA and fetal fraction quantification and be able to establish a normal range according to gestational age. The increase in cff-DNA suggests potential for a causative link between cff-DNA and spPTB. There are mechanisms by which DNA, and possibly cff-DNA, can be pro-inflammatory. It is however unclear how cff-DNA can reach the intracellular compartment where cell-sensing mechanisms (TLR9 and STING pathway) exist, and whether any consequent TLR9 and STING activation would elicit a response that can lead to spPTB. *In vivo* studies have given conflicting results and further investigation should be performed using cff-DNA from placental origin and in a manner that the potential pathway of inflammation can be identified. The effects of this pathway on PTB must then be established to be able to prove causative link between cff-DNA and PTB. As DNA can be pro-inflammatory and pregnancy yields in a unique type of cfDNA in the maternal circulation, more research is merited to further unravel the possible inflammatory role of cff-DNA in pregnancy and parturition.

## Declaration of interest

The authors declare that there is no conflict of interest that could be perceived as prejudicing the impartiality of the research reported.

## Funding

This research was made possible by funding from Tommy’s Baby Charity and the Medical Research Council Centre for Reproductive Health. Donald J Davidson was supported by a Medical Research Council Senior Non-clinical Fellowship (G1002046).
